# Target-based therapeutic matching of phase I trials in patients with metastatic breast cancer in a tertiary referral centre

**DOI:** 10.1038/s41416-018-0290-8

**Published:** 2018-10-15

**Authors:** Brent O’Carrigan, Joline Si Jing Lim, Awais Jalil, Samuel John Harris, Dionysis Papadatos-Pastos, Udai Banerji, Juanita Lopez, Johann Sebastian de Bono, Timothy Anthony Yap

**Affiliations:** 1 Drug Development Unit, Royal Marsden Hospital, London, UK; 2 National University Cancer Institute of Singapore, Singapore, Singapore; 3 Division of Clinical Studies, The Institute of Cancer Research, London, UK

**Keywords:** Cancer genomics, Predictive markers, Drug development

## Abstract

**Background:**

Greater understanding of the molecular classification of breast cancer has permitted the development of rational drug design strategies. In a phase I clinical trial setting, molecular profiling with next-generation sequencing of individual tumour samples has been employed to guide treatment.

**Methods:**

We conducted a retrospective evaluation of clinical outcomes of patients with metastatic breast cancer (MBC) treated in phase I clinical trials at our institution to assess the benefit of molecularly matched compared to non-matched treatments.

**Results:**

A total of 97 consecutive patients with MBC were enrolled onto ≥1 trial between 2009 and 2015. Fourteen patients participated in multiple trials, and a total of 113 trial encounters were reviewed in this retrospective study. Eighty-three percent of patients with molecular data available were able to participate in trials matched to molecular aberrations. Patients who were treated on matched studies had improved clinical benefit (RR: 1.80, *p* = 0.005), progression-free (HR: 0.52, *p* = 0.003) and overall survival (HR: 0.54, *p* < 0.001). Treatment was well tolerated with low rates of treatment discontinuation for toxicity (8% overall) that did not differ between groups. No toxicity-related deaths were observed.

**Conclusions:**

Molecular profiling for MBC patients in a phase I setting is feasible and aids therapeutic decisions with improved patient outcomes.

## Background

As our understanding of the molecular mechanisms underlying cancer progression continues to deepen, more molecular targeted agents are being rationally developed to potently target specific driver aberrations in tumours. In breast cancer, targeted therapies against mTOR (everolimus, Novartis) and CDK4/6 (palbociclib, Pfizer) have been granted regulatory approval for treatment, although predictive biomarker analyses have not demonstrated statistically significant correlations between candidate biomarkers and treatment outcomes.^1,2^ The use of molecular profiling of individual tumour samples from patients with breast cancers to guide treatment choice has now become more feasible with improved and more cost-efficient genomic next-generation sequencing (NGS) techniques, and studies have provided proof of concept that such a personalised approach is a rational strategy in cancer medicine and may lead to improved patient outcomes.^3,4^ In an early-phase clinical trial setting where there is a dearth of data in guiding treatment choice, molecular profiling may have an important role in guiding physicians in making rational treatment decisions based on the scientific knowledge of the underlying cancer biology matched with the molecular pharmacology of the antitumor agent.

In this retrospective study, we evaluated the clinical outcomes of patients with metastatic breast cancer (MBC) treated within a dedicated phase I clinical trials unit at our institution. The primary aim of the study was to assess the benefit of molecularly matched therapy compared to non-matched therapy, with secondary objectives of reporting the prevalence of molecular aberrations among these patients, as well as the safety and tolerability of these agents in a phase I setting.

## Methods

### Patient selection

This retrospective study included all patients with MBC treated on phase I clinical trials involving at least 1 novel molecular targeted agent at the Drug Development Unit, Royal Marsden National Health Service Foundation Trust, London, United Kingdom from 1 January 2009 to 31 December 2015. All patients provided written informed consent prior to trial enrolment, and all trials were approved by the Research Ethics Committee. Baseline data collected included patient demographics and prognostic variables, as well as clinical outcomes such as response rates (RR), progression-free survival (PFS) and overall survival (OS).

All patients included in this study were previously reviewed in the phase I clinical trials clinic to determine their suitability for study enrolment, including the assessment of their Eastern Co-operative Oncology Group (ECOG) performance status, overall organ function and overall patient interest in phase I clinical trial trials. Suitable patients were then offered participation into the Drug Development Unit Tissue Molecular Characterisation programme where targeted NGS was used to identify putative molecular aberrations in archived tumour samples. Fresh tumour tissue was collected for NGS analysis only if a contemporary tumour biopsy was mandated for specific trial enrolment. Germline *BRCA1/2* mutation sequencing was offered to patients in accordance with the clinical standard of care guidelines set forth by the UK National Institute for Clinical Excellence (NICE) or if it was required for phase I clinical trial entry, unless it had already been conducted prior to referral to the phase I clinical trial unit. Patients were discussed in a multi-disciplinary phase I clinical trials meeting comprised of tumour-specific oncologists, onco-geneticist, radiologists, nursing team, trial coordinators, data managers and laboratory technicians before allocation to a specific clinical trial was made, if appropriate. Trial allocation was determined based on scientific, preclinical and clinical evidence, including relevant clinical, pathologic and molecular data available for the individual patient. Patients entered on clinical trials were monitored and assessed using the Common Terminology Criteria for Adverse Events (CTCAE) version 4.0 for toxicities and laboratory variables.^5^ Patients had safety evaluations weekly, and tumour response assessments after every two treatment cycles, using computed tomography scans evaluated by Response Evaluation Criteria in Solid Tumours (RECIST) criteria version 1.1.^6^

### Genomic analysis

Somatic targeted amplicon NGS was conducted at the Institute for Cancer Research on patients who provided informed consent and who had archival and/or fresh tumour tissue available.^7^ All tumour biopsy samples were evaluated using haematoxylin and eosin staining for tumour cellularity and marked for coring. DNA was manually extracted using the QIAamp DNA FFPE Tissue kit (Qiagen, Venlo, Limburg, Netherlands) following the manufacturer’s protocol. Eluted DNA was measured using nanodrop and Quant-iT high-sensitivity Picogreen double-stranded DNA (dsDNA) Assay Kit (Invitrogen, ThermoFisher Scientific Corp., Waltham, MA, USA), according to the manufacturer’s recommendations. DNA quality control methods and criteria have been published previously.^7^

Libraries were constructed with the use of the TruSeq Amplicon Cancer Panel covering 212 regions of interest in 48 cancer-related genes (Supplementary Table 1) and run on a MiSeq sequencer (Illumina) according to the manufacturer’s protocol. From October 2015, this panel was expanded to a 113 gene panel with the GeneRead DNAseq Damage Panel (DDP-V2, Qiagen) (Supplementary Table 2). Bioinformatic analyses were performed utilising the MiSeq Reporter Software MCS 2.2.0, RTA 1·17·28·0 and Nextgene (from Biogene, Kimbolton, Cambs, UK).

### Definition of matched trial

A trial encounter was defined as matched if molecular analysis revealed a potentially actionable aberration required for trial eligibility (genotype-selected), or if a patient harboured a potentially actionable somatic or germline mutation that was within the pathway targeted by the study drug (genotype-relevant). Trial encounters were considered non-matched if no molecular aberrations were available, or aberrations identified were not considered actionable within the portfolio of prevalent clinical trials.

### Statistical analysis

Patient characteristics were summarised with descriptive statistics. OS was defined as the interval between the first administration of the study agent and the date of death from any cause. Patients who were lost to follow-up were censored at the date of last contact. PFS was defined by the time elapsed between the date of first administration of study agent and radiological progression or death from any cause (whichever occurred first). If no evidence of disease progression was documented at the last follow-up, patients were censored at the time of last radiological evaluation. Median PFS and OS were estimated using the Kaplan–Meier method, and survival function between groups were compared with a two-sided log-rank test. Clinical benefit rate (CBR) was defined as the sum of confirmed RECIST partial response (PR), complete response (CR) and stable disease (SD) ≥ 8 weeks. All *p*-values presented in this study are two-sided. All analyses were performed using GraphPad Prism v6.0.

## Results

### Baseline patient and tumour characteristics

A total of 97 consecutive patients with MBC who participated in at least one phase I trial between 2009 and 2015 were included in this retrospective study. Fourteen patients participated in multiple phase I trials, and a total of 113 trial encounters were reviewed in this study. Overall, the median age was 52.1 years (range: 27–-92 years). 47% (53/113) of patients had oestrogen receptor (ER) and/or progesterone receptor (PR) positive, HER2-negative disease; 19% (22/113) had HER2-positive disease; and 34% (38/113) had triple-negative breast cancer (TNBC). Prior lines of therapy included chemotherapy (median 3, range: 0–8), endocrine (median 2, range: 0–4) and HER2-directed therapies (median 2, range: 1–4). The median number of disease sites was 3 (range: 1–7), with bony metastases being the most common distant metastatic site (53%), followed by lung (39%) and liver (34%) metastases. Two patients (2%) had brain metastases at enrolment, both of whom had HER2-positive disease. The baseline characteristics of patients enrolled in matched and non-matched trials were similar, with no significant differences observed (Table 1).

**Table 1:** Baseline demographics and patient characteristics

Baseline characteristics	Matched (*N* = 59)	Non-matched (*N* = 54)
Age, mean (SD), years	51.1 (12.7)	51.5 (10.1)
ECOG PS baseline, *n* (%)		
0	36 (61)	33 (61)
1	23 (39)	21 (39)
RMH prognostic score, *n* (%)		
0–1	33 (56)	34 (63)
2–3	26 (44)	20 (37)
Histologic subtype, *n* (%)		
ER+/HER2−	27 (47)	24 (47)
TNBC	21 (35)	17 (32)
HER2+/ER+	8 (13)	7 (12)
HER2+/ER−	3 (5)	6 (11)
Number sites of disease		
Median (range)	3 (1–7)	3 (1–6)
Sites of disease, *n* (%)		
Nodal	41 (68)	29 (55)
Locoregional	29 (48)	37 (70)
Bone	35 (58)	25 (47)
Lung	26 (43)	18 (34)
Liver	18 (30)	20 (38)
CNS	0 (0)	2 (4)
Lines of prior therapy, median (range)		
Chemotherapy	3 (0–8)	3 (0–8)
Endocrine therapy^a^	2 (1–4)	2 (1–4)
HER2-directed therapy^b^	2 (1–4)	2 (1–4)

*CNS* Central nervous system, *ECOG* Eastern Co-operative Oncology Group, *ER* oestrogen receptor, *HER2* human epidermal growth factor receptor 2, or erbB-2, *PS* performance status, *RMH* Royal Marsden Hospital, *SD* standard deviation, *TNBC* triple-negative breast cancer

^a^Only participants with ER+ MBC

^b^Only participants with HER2+ MBC

### Molecular testing

**Fig. 1:**
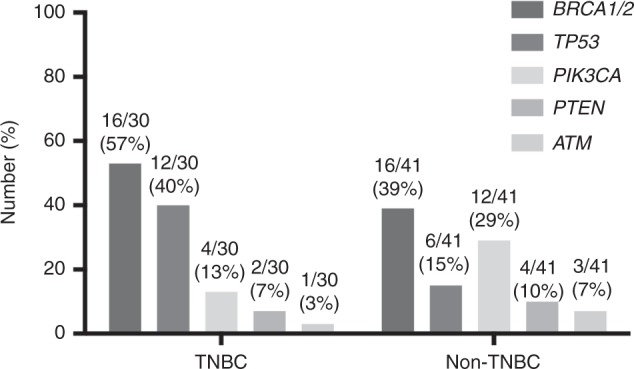
Mutational landscape from targeted amplicon NGS profiling of patients differs by histologic subtype. The number and proportion of participants with mutations identified in the listed genes are shown by histologic subtype TNBC and non-TNBC. TNBC triple-negative breast cancer

Of 113 trial encounters, 71 (63%) had molecular data available from germline *BRCA1/2* mutation and/or somatic NGS testing. Germline *BRCA1/2* mutations were observed in 28% (32/113) of trial encounters. Molecular aberrations along the PI3K/AKT/mTOR pathway were noted in 23% (26/113) of encounters, including *PI3KCA* (*n* = 16), *AKT* (*n* = 3) and *PTEN* mutations (*n* = 10). Other mutations of interest include *TP53* (16%, 18/113) and *ATM* (4%, 5/113). The mutational landscape differed by breast cancer subtype with common mutations detected in patients with advanced TNBC (n = 30) being *BRCA1/2* (57%, 16/30), *TP53* (40%, 12/30), and *PIK3CA* (13%, 4/30). In HR and/or HER2-positive MBC (*n* = 41), *BRCA1/2* (39%, 16/41), *PI3KCA* (29%, 12/41) and *TP53* (15%, 6/41) mutations were most commonly identified (Fig. 1). The distribution of *BRCA1/2* mutations identified differed by breast cancer subtype, with *BRCA1* mutations predominated among TNBC (94%, 15/16) while *BRCA2* mutations predominated among patients with HR and/or HER2-positive MBC (75%, 12/16).

### Allocation of trial therapy

Overall, 52% (59/113) of trial encounters were matched with patients harbouring a molecularly selected tumour, while 48% (54/113) of encounters were considered non-matched. Among encounters with available molecular data, 83% (59/71) were successfully matched. Encounters were non-matched either because no molecular data were available (78%, 42/54); or even though such data were available, there were no suitable matched trials (22%, 12/54), that is, no trial slots were available within a clinically appropriate time.

A total of 97 patients with MBC were enrolled onto 33 different phase I clinical trials, with therapeutic targets that included poly(ADP-ribose) polymerase (PARP), androgen receptor (AR), PI3K, mammalian target of rapamycin complex (mTORC) 1/2, heat shock protein (HSP) 90, AKT, Rho-associated protein kinase (ROCK) 1/2, B-cell lymphoma (BCL) 2, PIM kinase, and HER2. Eleven patients were treated with a combination of two agents, including a combination of PARP with AKT inhibitor (*n* = 5), dual PI3K/mTOR kinase inhibitor (*n* = 3), AKT with MEK inhibitor (*n* = 2), and AKT with PI3K inhibitor (*n* = 1).

Of the 97 patients, 14 participated in ≥2 trials, while two patients participated in ≥3 trials. There was no significant difference between patients participating in multiple compared to single trials. Participants of multiple compared to single trials had similar age (median 51.9 vs 51.1 years), but numerically poorer performance status (ECOG ECOG 0: 29% vs 41%, *p* = 0.38), greater disease burden (median 3 vs 2 disease sites), more likely to have TNBC histology (43% vs 29%, *p* = 0.29) and harbour a *BRCA1/2* mutation (36% vs 24%, *p* = 0.35). Trial encounters from patients participating in multiple trials (*n* = 30) were well balanced between matched (57%, 17/30) and non-matched (43%, 13/30). Participants of multiple trials were not more likely to participate in trials of any particular class of agents. For example, despite a numerical enrichment for TNBC histology and/or *BRCA1/2* mutations among participants of multiple trials, such participants were not more likely overall to accrue to trials of PARP inhibitors or combinations (17%, 5/30) than participants of single trials (31%, 26/83, *p* = 0.15, Fisher’s exact test).

Among patients enrolled on matched clinical trials (*n* = 59), 34% (20/59) were matched to single agent PARP inhibitor, 29% (17/59) to AR inhibitor monotherapy, 19% (11/59) to a dual PI3K/mTOR inhibitor, 7% (4/59) to a combination of PARP and AKT inhibitors, 7% (4/59) to HER2-directed therapy and 4% (3/59) to other matched treatments. Among patients enrolled on non-matched clinical trials (*n* = 54), the majority of patients were enrolled onto the dual PI3K/mTOR inhibitor study (54%, 29/54); 13% onto studies involving at least 1 PARP inhibitor (11% on PARP inhibitor monotherapy, 2% on the combination of a PARP inhibitor and AKT inhibitor), and 33% (18/54) were enrolled on inhibitors against AKT (*n* = 8), HSP90 (*n* = 6), pan-PIM (*n* = 1), BCL2 (*n* = 1), and combination studies of AKT and MEK inhibitors (*n* = 1), and AKT and PI3K inhibitors (*n* = 1) (Table 2).

**Table 2:** Trial allocation

Trial mechanism pathway	Matched (*N* = 59) *n* (%)	Non-matched (*N* = 54) *n* (%)
PARP inhibitors	20 (34)	6 (11)
BRCA1	12	
BRCA2	8	
AR inhibitors	17 (29)	—
AR+	10	
ER+	7	
PI3K/mTOR inhibitors	11 (19)	29 (54)
PIK3CA	8	
AKT	2	
PTEN	1	
PARP + AKT inhibitors	4 (7)	1 (2)
BRCA1	2	
BRCA2	2	
Anti-HER2 therapies	4 (7)	—
Other inhibitors	3 (4)	18 (33)
AKT	1	8
HSP90	1	6
ATR	1	—
AKT + MEK	—	1
AKT + PI3K	—	1
BCL2	—	1
Pan-PIM	—	1

*AKT* Protein kinase B, *ATR* ataxia telangiectasia and Rad3-related protein, *AR* androgen receptor, *BCL2* B-cell lymphoma 2 protein, *ER* oestrogen receptor, *HER2* human epidermal growth factor receptor 2, or c-erbB-2, *HSP90* heat shock protein 90, *mTOR* mammalian target of rapamycin, *PARP* poly (ADP-ribose) polymerase, *PI3K* phosphatidylinositol-4,5-bisphosphate 3-kinase, *PTEN* phosphatase and tensin homolog

### Clinical outcomes of phase I trials

Patients who were enrolled on phase I clinical trials tolerated treatment generally well. Rates of discontinuation due to drug-related toxicities were low (8%, 9/113) and did not differ between matched and non-matched arms (*p* = 0.83). No deaths due to drug-related toxicities were observed.

Of 113 trial encounters, 107 were evaluable for antitumor response assessment. The best overall response was confirmed RECIST partial response (PR) in 17% (18/107) patients, and prolonged stable disease (SD ≥ 8 weeks) in 32% (34/107) patients, for an overall clinical benefit rate (CBR, sum PR and prolonged SD) of 45% (48/107). Overall median PFS was 2.2 months (range: 0.3–14.3 months) and median OS was 7.2 months (range: 0.4–58 months).

**Fig. 2:**
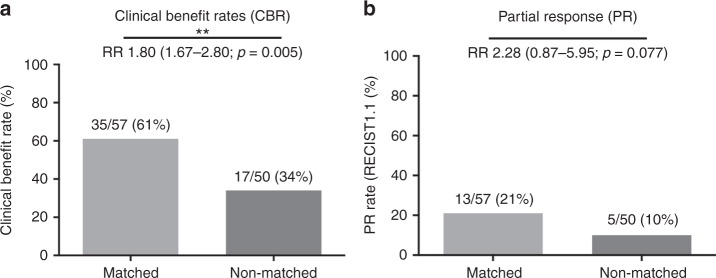
**a** Clinical benefit rate (CBR) and **b** partial response (PR) rate among participants on matched vs non-matched trials. Two-sided *χ*^2^ test

**Fig. 3:**
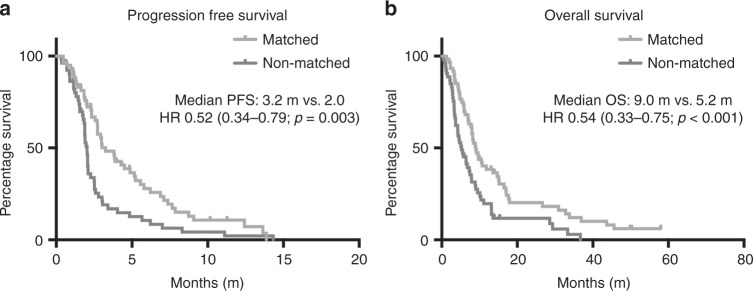
**a** Progression-free survival (PFS) and **b** overall survival (OS) among participants of matched vs non-matched trials from the first administration of study agent for each trial encounter. Kaplan–Meier survival analysis with log-rank (Mantel–Cox) test and hazard ratio (HR)

Overall outcomes were improved when patients were treated on matched vs unmatched trials. Patients on matched trials had a significantly improved CBR (61% vs 34%, RR: 1.80, *p* = 0.005), and a trend towards improved PR rates (21% vs 10%, RR: 2.28, *p* = 0.077) (Fig. 2). This was also associated with improved median PFS (3.2 vs 2 months, HR 0.52, *p* = 0.003) and improved median OS (9 vs 5.2 months, HR: 0.54, *p* < 0.001) (Fig. 3).

## Discussion

Our study of patients with MBC treated in a phase I clinical trial setting reported results consistent with previous publications.^8^ When molecular data were available, a large proportion of patients were able to undergo successful matching (83%), and patients treated on matched studies had improved patient outcomes. Compared to patients who were treated on non-matched studies, patients enrolled on matched studies experienced greater clinical benefit (RR: 1.80, *p* = 0.005), with improvement in both median PFS (HR: 0.52, *p* = 0.003) and median OS (HR: 0.54, *p* < 0.001). Treatment was generally well tolerated, with only 8% of patients discontinuing due to drug-related toxicities, and no deaths due to drug toxicities were observed. Our study also showed that there is a high prevalence of actionable aberrations within an enriched MBC phase I trial population. This suggests that molecular profiling may be helpful in identifying potential therapeutic options for patients and should be considered for all patients with refractory breast cancers for which standard therapeutic options have been exhausted.

Breast cancer is a heterogeneous disease, with recent advances in genomic analysis allowing greater resolution of molecularly discrete subtypes.^9–11^ While breast cancer has been conventionally divided into subtypes based on histological findings, including cell origin, ER, PR and HER2 receptor status, significant heterogeneity in prognosis and treatment outcomes has been observed. With the advent of genomic profiling, there is increasing evidence that molecularly distinct phenotypes may better reflect tumour activity and clinical responses compared to conventional histological classification.^9–11^ Apart from somatic mutations, other aberrations including genome rearrangements may also be of significance and provide further insights into the underlying biology of breast cancer.^12^

Despite substantive advances in understanding the heterogeneity in breast cancer biology, translation of these findings into clinically meaningful therapeutic options have been limited to a few successes. Classical molecular biomarkers such as HER2^13,14^ and ER expression^15^ have provided the largest clinical evidence base. In addition, the PARP inhibitor olaparib (AstraZeneca) was recently shown to result in significant clinical benefit over standard therapy in patients with advanced *BRCA1/2* mutant cancers, including median progression-free survival, leading to Food and Drug Administration (FDA) approval. Retrospective analyses of patients with advanced HER2-positive breast cancers treated with everolimus in the context of the BOLERO-1 and BOLERO-3 trials have suggested a correlation between patients with a hyperactive phosphatidylinositol-3-kinase (PI3K) pathway and PFS benefit,^16^ although such a correlation was not observed in patients with advanced HER2-negative breast cancers treated with everolimus in the context of the BOLERO-2 study.^17^

Upon exhaustion of conventional treatments, appropriate patients are commonly referred to phase I clinical trial units for consideration of novel antitumor agents. In the era of precision medicine, large-scale genomic testing is increasingly employed in phase I settings to facilitate enrolment onto genomically matched studies.^4^ A phase I clinical trial unit provides a unique setting where patients can be treated with novel antitumor agents using such molecular data, although clinical qualification of the preliminary results will ultimately still be required in late phase clinical trials with larger patient populations. A meta-analysis of 346 published phase I studies involving patients with advanced solid tumours reported that patient outcomes were improved when a biomarker-based selection strategy was employed, with improved response rates (RR: 30.6% vs 4.9%, *p* < 0.0001) and PFS (5.7 vs 2.95 months, *p* = 0.0002).^18^ A previous retrospective review limited to patients with advanced triple-negative breast cancers (TNBC) treated in a phase I clinical trial setting reported a clinical benefit rate (CBR) of 12%, with patients treated on matched therapies shown to have improved outcomes compared to those receiving non-matched therapies.^19^

There are several limitations with our study. Retrospective analyses are prone to selection and other forms bias, but our data provide several points of reassurance. Characteristics of participants of multiple vs single trials were specifically compared to ensure that young, fit participants with higher performance status and low disease burden, that might permit enrolment in multiple trials, were not biasing the results towards matched trials. This is not the case as no significant difference in patient characteristics was observed, with numerically poorer PS and greater disease burden observed among participants of multiple trials. Participants of multiple trials were not more likely to be allocated to matched trials, nor a particular therapeutic class suggesting the observed clinical benefits of target-based therapeutic matching extends beyond the spectrum of clinically well described drugs such as PARP inhibitors.

An additional explanation of the improved outcomes in the matched cohort is the differential allocation of breast cancer subtypes based on available biomarker assays. For example, patients allocated to treatment with AR inhibitors (29% of the matched cohort) included patients with TNBC with AR expression detected using an immunohistochemistry assay. This cohort is known to have better survival than non-AR-expressing TNBC.^20,21^

Intratumor heterogeneity and cancer evolution over multiple lines of antitumor therapies have been well documented,^22^ and the lack of routine fresh tissue biopsies for molecular profiling may confound the genomic data yielded from NGS. While efforts were made to obtain fresh tumour tissue, the majority of patients had molecular profiling performed on archived tumour tissue obtained from their initial primary tumour resection, and thus, may not be an accurate reflection of the patient’s current mutational profile. Although NGS was carried out on all patients with available tumour tissue who consented to molecular profiling, two different panels were utilised. While the newer panel covered an expanded 113 genes, including an increased number of driver aberrations involved in key pathways, such as DNA damage repair, the previous panel comprising 48 genes may have underestimated the number of potentially actionable mutations present. Additionally, while patients were matched to genotype-relevant trials based on our current scientific understanding of the underlying tumour biology, it remains challenging to be certain that the detected aberrations targeted are truncal driver mutations rather than sub-clonal mutations.

In conclusion, our study suggests that the molecular profiling for patients with MBC in a phase I setting is feasible and may help to direct therapeutic decisions. Patients who are matched to genotype-selected or genotype-relevant trials have superior clinical outcomes, in terms of CBR, PFS and OS, with good tolerability.

## Electronic supplementary material

Supplementary material

## Data Availability

Requests for data are encouraged and will be considered by the authors.
