# Supplementary material for: Target-based therapeutic matching of phase I trials in patients with metastatic breast cancer in a tertiary referral centre
Source: Br J Cancer. 2018 Oct 15;119(8):922–7. doi: 10.1038/s41416-018-0290-8 (PMC6203714; doi:10.1038/s41416-018-0290-8)
Supplement: Supplementary file 1 — Supplementary material [file 41416_2018_290_MOESM1_ESM.pdf]

Supplementary Appendix

Brent O'Carrigan, Joline SJ Lim, Awais Jalil, Samuel J Harris, Dionysis Papadatos-Pastos, Udai Banerji, Juanita Lopez, Johann S de Bono, Timothy A Yap

Target-based therapeutic matching of phase I trials in patients with metastatic breast cancer in a tertiary referral centre

Table 1: 48 gene Tru-Seq Amplicon Cancer Panel (Illumina)
Jan 2009 – Sept 2015

ABL1	**JAK2**
**AKT1**	JAK3
**ALK**	KDR
**APC**	KIT
**ATM**	**KRAS**
**BRAF**	**MET**
**CDH1**	**MLH1**
**CDKN2A**	MPL
CSF1R	**NOTCH1**
**CTNNB1**	NPM1
**EGFR**	**NRAS**
**ERBB2**	**PDGFRA**
**ERBB4**	**PIK3CA**
FBXW7	**PTEN**
FGFR1	PTPN11
**FGFR2**	**RB1**
**FGFR3**	**RET**
FLT3	SMAD4
GNA11	**SMARCB1**
GNAQ	SMO
GNAS	**SRC**
**HNF1A**	**STK11**
**HRAS**	**TP53**
IDH1	**VHL**

Note: Gene covered by both NGS panels

Table 2: 113 gene GeneRead DNA Damage Panel (Qiagen)
Oct – Dec 2015

**AKT1**	CHEK2	FANCM	MYD88	SMARCA4
AKT2	**CTNNB1**	**FGFR2**	NBN	**SMARCB1**
**ALK**	DDB2	**FGFR3**	NF1	SPOP
**APC**	**EGFR**	**HNF1A**	NF2	**SRC**
AR	EPCAM	**HRAS**	NFKBIA	**STK11**
ARID1A	**ERBB2**	JAK1	**NOTCH1**	TNFAIP3
ARID2	ERBB3	**JAK2**	NOTCH2	TNFRSF14
**ATM**	**ERBB4**	**KRAS**	**NRAS**	**TP53**
ATR	ERCC2	MAP2K1	NTRK1	TSC1
ATRX	ERCC3	MAP2K2	PALB2	TSC2
AXIN1	ERCC4	MAP2K4	**PDGFRA**	**VHL**
AXIN2	ERCC5	MAP3K1	**PIK3CA**	WRN
BARD1	ERCC6	MAP4K3	PIK3CG	WT1
BLM	EZH2	MDM2	PIK3R1	XPA
**BRAF**	FAM46C	**MET**	PMS2	XPC
BRCA1	FANCA	**MLH1**	PRKDC	XRCC3
BRCA2	FANCB	MLH3	**PTEN**	ZRSR2
BUB1B	FANCC	MRE11A	RAD50	
**CDH1**	FANCD2	MSH2	RAD51B	
CDK12	FANCE	MSH3	RAD51C	
CDK4	FANCF	MSH6	RAD51D	
CDKN1B	FANCG	MTOR	**RB1**	
**CDKN2A**	FANCI	MUTYH	RECQL4	
CHEK1	FANCL	MYC	**RET**	

Note: Gene covered by both NGS panels
